# D-dimer, CRP, PCT, and IL-6 Levels at Admission to ICU Can Predict In-Hospital Mortality in Patients with COVID-19 Pneumonia

**DOI:** 10.1155/2022/8997709

**Published:** 2022-02-28

**Authors:** Marija Milenkovic, Adi Hadzibegovic, Mirjana Kovac, Bojan Jovanovic, Jovana Stanisavljevic, Marina Djikic, Djuro Sijan, Nebojsa Ladjevic, Ivan Palibrk, Marija Djukanovic, Jelena Velickovic, Sanja Ratkovic, Milica Brajkovic, Viseslav Popadic, Slobodan Klasnja, Borislav Toskovic, Darko Zdravkovic, Bogdan Crnokrak, Olivera Markovic, Jelica Bjekic-Macut, Aleksandra Aleksic, Simona Petricevic, Lidija Memon, Ana Milojevic, Marija Zdravkovic

**Affiliations:** ^1^University Clinical Centre of Serbia, Belgrade, Serbia; ^2^Faculty of Medicine, University of Belgrade, Belgrade, Serbia; ^3^Blood Transfusion Institute of Serbia, Belgrade, Serbia; ^4^University Clinical Hospital Center Bezanijska Kosa, Belgrade, Serbia

## Abstract

**Introduction:**

Health care workers have had a challenging task since the COVID-19 outbreak. Prompt and effective predictors of clinical outcomes are crucial to recognize potentially critically ill patients and improve the management of COVID-19 patients. The aim of this study was to identify potential predictors of clinical outcomes in critically ill COVID-19 patients.

**Methods:**

The study was designed as a retrospective cohort study, which included 318 patients treated from June 2020 to January 2021 in the Intensive Care Unit (ICU) of the Clinical Hospital Center “Bezanijska Kosa” in Belgrade, Serbia. The verified diagnosis of COVID-19 disease, patients over 18 years of age, and the hospitalization in ICU were the criteria for inclusion in the study. The optimal cutoff value of D-dimer, CRP, IL-6, and PCT for predicting hospital mortality was determined using the ROC curve, while the Kaplan-Meier method and log-rank test were used to assess survival.

**Results:**

The study included 318 patients: 219 (68.9%) were male and 99 (31.1%) female. The median age of patients was 69 (60-77) years. During the treatment, 195 (61.3%) patients died, thereof 130 male (66.7%) and 65 female (33.3%). 123 (38.7%) patients were discharged from hospital treatment. The cutoff value of IL-6 for in-hospital death prediction was 74.98 pg/mL (Sn 69.7%, Sp 62.7%); cutoff value of CRP was 81 mg/L (Sn 60.7%, Sp 60%); cutoff value of procalcitonin was 0.56 ng/mL (Sn 81.1%, Sp 76%); and cutoff value of D-dimer was 760 ng/mL FEU (Sn 63.4%, Sp 57.1%). IL-6 ≥ 74.98 pg/mL, CRP ≥ 81 mg/L, PCT ≥ 0.56 ng/mL, and D-dimer ≥ 760 ng/mL were statistically significant predictors of in-hospital mortality.

**Conclusion:**

IL-6 ≥ 74.98 pg/mL, CRP values ≥ 81 mg/L, procalcitonin ≥ 0.56 ng/mL, and D-dimer ≥ 760 ng/mL could effectively predict in-hospital mortality in COVID-19 patients.

## 1. Introduction

In December 2019, SARS-CoV-2 was identified for the first time as a cause of COVID-19 disease by Chinese scientists [1]. However, after more than a year since the pandemic's beginning, we still do not have a complete picture of the disease itself.

Initially, COVID-19 was considered a respiratory disease, with pneumonia being the most common and deadliest complication. However, SARS-CoV-2 has been shown to trigger an excessive and uncontrolled immune-hemostasis response that causes many complications, such as thrombosis, tissue damage, ARDS, DIC, and MODS; therefore, not only it is necessary to understand COVID-19 as a respiratory, but also as a potential multisystem disease [2, 3].

Studies from the beginning of pandemic estimated overall hospital mortality from COVID-19 are approximately 15% to 20%, but up to 40% among patients requiring ICU admission; however, mortality rates vary across age cohorts, from 5% among patients younger than 40 years to greater than 60% for patients aged 80 to 89 years [4]. In contrast, a recent study suggested lower mortality due to the presence of appropriate treatment and vaccination [5].

It is well known that thrombosis is an important complication that significantly increases the risk of a deadly outcome. A great number of thrombosis was verified in SARS-COV2-positive patients [6–8]. Even, despite standard thromboprophylaxis doses of LMWH, 31% of individuals with proven COVID-19 pneumonia developed thrombosis in ICU [9]. Significant changes in coagulation parameters were verified in patients with COVID-19. Concerning the patients, higher values of D-dimer were observed in persons requiring treatment in ICU [10]. Consequently, higher D-dimer values were associated with severe clinical presentation of COVID-19 disease, as mentioned above [11–14].

Considering all of this, D-dimer and other inflammatory parameters such as IL-6, CRP, and PCT might be used to predict mortality. Predictors of mortality among laboratory parameters are important as they can reflect possible mechanisms of disease progression and give important information on potentially useful therapeutic modalities [15]. Adequate and precise predictors are crucial, especially in the pandemics era.

The aim of this study was to identify potential biochemical predictors of in-hospital mortality among COVID-19 patients and to determine their predictive cutoff values.

## 2. Methods

### 2.1. Study Design and Participants

The study was designed as a retrospective cohort study, which included 318 patients treated from June 2020 to January 2021 in the Clinical Hospital Center “Bezanijska Kosa” ICU in Belgrade. The criteria for inclusion in the study were the verified diagnosis of COVID-19 disease, patients over 18 years of age, and hospitalization in the ICU. The criteria for excluding patients from the study were incomplete data, the patient's stay in the ICU for some other reasons not due to complications of COVID-19 (e.g., postoperative treatment of patients), and transfer of patients to other medical institutions for COVID-19 treatment.

### 2.2. Definitions, Diagnosis, and Outcomes

COVID-19 diagnosis was made based on the clinical symptoms and signs of the disease, with/without a positive radiological finding (X-ray, CT), and a positive result of the nasopharyngeal swab SARS-CoV-2, detected by the RT-PCR method. The main clinical criteria for Respiratory ICU admission was radiographic or CT scan severity score progression, peripheral oxygen saturation (Sp02) below 93% despite maximal conventional supportive oxygen therapy (up to 15 L/min through a nasal cannula, conventional oxygen, or nonrebreather mask), laboratory test results, mainly an increase of inflammatory parameters after repeated controls, and arterial blood gas test. Critically ill patients on invasive, noninvasive ventilation and high flow oxygen therapy with moderate and severe ARDS were selected for the study according to the Berlin definition of ARDS [16]. The primary outcome of interest was in-hospital mortality and was stratified as deceased or discharged from the hospital. Survivors refer to participants who were discharged from the hospital, and no survivors refer to deceased participants. All patients were followed until their outcomes.

### 2.3. Treatment

During the hospitalization, patients were treated according to the adjusted National protocol of the Republic of Serbia to treat COVID-19 infection [17]. Antiviral agents (favipiravir, remdesivir) were used 5-7 days from symptom onset in patients on supportive oxygen therapy and with radiographically verified severe bilateral pneumonia. Corticosteroids (prednisone 0.5 mg/kg in two doses, methylprednisolone 1-2 mg/kg, and dexamethasone 6 mg/day) were used in patients with moderate to severe clinical image with signs of clinical deterioration or in patients with incipient or developed ARDS. Anticoagulant therapy was used in the standard prophylactic dose of LMWH for patients with multiple risk factors and conventional oxygen therapy. According to the anti-Xa levels, therapeutical doses were used for patients in the ICU requiring mechanical ventilation or high-flow oxygen therapy, those on long-term anticoagulant therapy, or those with suspectable or confirmed thrombosis. Antibiotics were used empirically or according to the antibiogram. The main criteria for tocilizumab administration were an increase in IL-6 values above 40 pg/mL and CRP values above 50 mg/L or a threefold increase during the last 48 h in patients with clinical worsening with more than 25 resp/min, saturation below 93%, and partial presure of oxygen below 8.66 kPa without supportive oxygen therapy. Convalescent plasma was used in patients with rapid worsening, positive PCR test for SARS-CoV-2 virus, in the first two weeks from symptom onset. The indication was established according to the specific scoring system with different variables, including the patient's clinical status, a form of the disease, time from symptom onset, respiratory status, radiographic findings, comorbidities, and applied therapy. Inotropic agents, noradrenaline, dobutamine, vasopressin, and adrenaline were used in a standard dosage.

### 2.4. Data Collection

The necessary data were obtained from the health information system of the Clinical Hospital Center “Bezanijska Kosa” (Heliant, v7.3, r48602). The data includes demographic data (age, gender), laboratory values (IL-6, CRP, PCT, ferritin, D-dimer, lymphocytes, thrombocytes, PT, aPTT, and fibrinogen), and the outcome of the treatment. Past medical history (hypertension, diabetes mellitus, COPD, coronary heart disease, obesity, heart failure, cardiomyopathy, and chronic kidney disease) was obtained from participants' medical documentations and was filed in the health information system. Clinical and laboratory parameters were followed upon admission to the hospital and ICU, with specific parameters followed during hospitalization.

### 2.5. Statistical Analysis

Descriptive statistics methods were used to process and present the results. Continuous variables were presented as the median and IQR and as the frequency (%) for categorical variables. The Mann–Whitney *U* test and Pearson's chi-square test were used to compare the data. The optimal cutoff value of D-dimer, CRP, IL-6, and PCT for predicting hospital mortality was determined using the ROC curve, while the Kaplan-Meier method and log-rank test were used to assess survival. A value of *p* < 0.05 was considered statistically significant.

## 3. Results

The study included 318 patients, 219 (68.9%) male and 99 (31.1%) female. The median age of patients was 69 (60-77) years. During the treatment, 195 (61.3%) patients died: thereof 130 were male (66.7%), and 65 were female (33.3%). 123 (38.7%) patients were discharged from the treatment. Age, gender, comorbidities, laboratory parameters, and CT score of patients are shown in Table 1.

*C*-indices for IL-6, CRP, PCT, and D-dimer are presented in Table 2. PCT has the highest *C*-index (0.77) to predict in-hospital mortality in COVID-19 patients.

**Figure 1:**
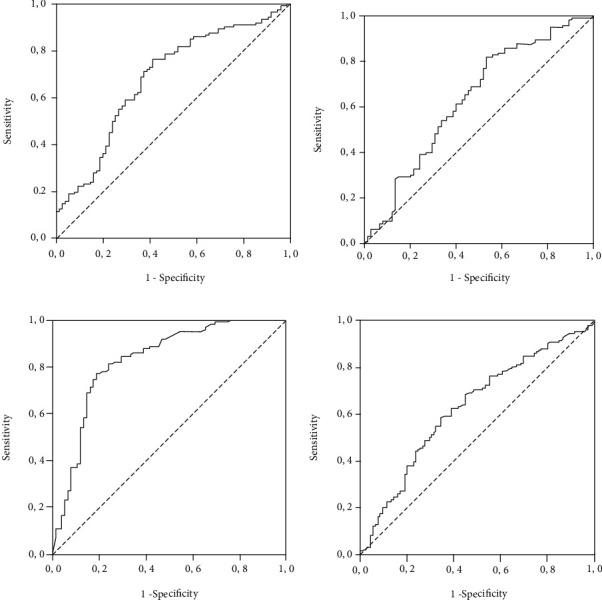
Receiver operator characteristic curve for (a) IL-6, (b) CRP, (c) PCT, and (d) D-dimer to predict deaths. The optimum cutoff point, identified as the point closest to the upper left corner, was for IL-6 (74.98 pg/mL), CRP (81 mg/L), PCT (0.56 ng/mL), and D-dimer (760 ng/mL FEU).

Cutoff values of the analyzed parameters were obtained using the ROC curve. The cutoff value of IL-6 for in-hospital death prediction was 74.98 pg/mL (Sn 69.7%, Sp 62.7%); cutoff value of CRP was 81 mg/L (Sn 60.7%, Sp 60%); cutoff value of PCT was 0.56 ng/mL (Sn 81.1%, Sp 76%); and cutoff value of D-dimer was 760 ng/mL FEU (Sn 63.4%, Sp 57.1%). ROC curves are presented in Figure 1.

**Figure 2:**
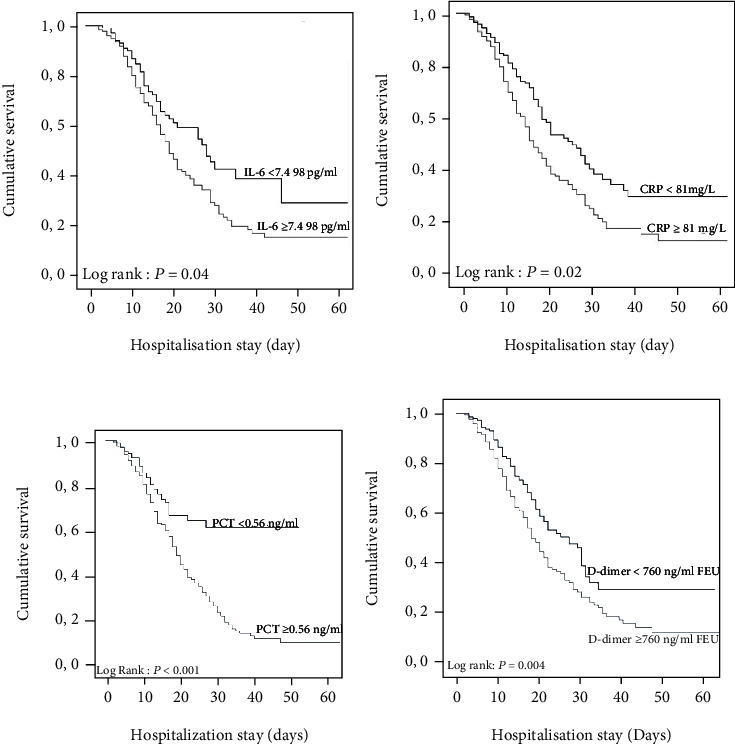
Kaplan-Meier survival curves for (a) IL-6, (b) CRP, (c) PCT, and (d) D-dimer levels on admission to the ICU.

Using the Kaplan-Meier survival curve and log-rank test, it was shown that IL-6 higher or equal to 74.98 pg/mL was a statistically significant predictor of in-hospital mortality (*p* = 0.04). In addition, CRP values higher or equal than CRP 81 mg/L, PCT higher or equal than 0.56 ng/mL, and D-dimer higher or equal than 760 ng/mL FEU represent significant predictors of in-hospital mortality (CRP, *p* = 0.02; PCT, *p* < 0.001; and D-dimer, *p* = 0.04) (Figure 2).

## 4. Discussion

First, our National protocol is mainly following the WHO treatment guidelines [18]. However, we would like to address a few differences between our National protocol and the WHO treatment guidelines. According to our National protocol, the main difference is the usage of favipiravir and remdesivir. The use of systemic corticosteroids, monoclonal antibodies, and IL-6 receptor blockers was in accordance with the WHO treatment guidelines. This slight discordance between protocols should not affect the discussion of our results with results in the literature.

The study indicated significant disorders of laboratory parameters in patients with COVID-19 treated in ICU. Elevated levels of IL-6, CRP, PCT, D-dimer, and lower serum albumin levels were detected in subjects with fatal disease outcomes during treatment. Significantly higher in-hospital mortality was observed in individuals whose IL-6 values were equal to or higher than 74.98 pg/mL, followed by CRP values higher than 81 mg/L, PCT values equal to or higher than 0.56 ng/mL, and D-dimer values equal to or higher than 760 ng/mL FEU. However, 5 out of 318 participants with IL-6, CRP, PCT, and D-dimer values above cutoff value survived. These findings suggest a good prediction of in-hospital mortality in patients with COVID-19 who require admission to the ICU, especially when jointly using all four cutoff values.

The cytokine storm is one of the most critical factors contributing to COVID-19 mortality. Elevated values of various cytokines, such as IL-1, IL-2, IL-6, IL-7, IL-8, IL-12, IFN, MCP-1, and TNF-*α*, were observed. In SARS-CoV-2-positive patients, cytokine storm is characterized by high serum concentrations of IL-6 and TNF-*α* predominantly [19, 20]. Our study observed higher mortality in patients with IL-6 concentrations higher than 74.98 pg/mL. In addition, other studies also favoured a more severe form of the disease and higher mortality of patients with higher values of IL-6 [21–23]. Patients whose maximum IL-6 values exceeded 80 pg/mL had a significantly higher probability of need of invasive mechanical ventilation. Hyperinflammatory response in the setting of COVID-19 could also be responsible for the potential multiorgan failure and various life-threatening complications, including ARDS, myocardial damage, and kidney and liver failure. Also, a significant predictor was elevated values of CRP [24].

In addition to IL-6, CRP is a significant marker of COVID-19 inflammation. Higher levels of serum CRP are associated with higher mortality in people with severe COVID-19 disease [25], more specifically, CRP values above 77.35 mg/L [26]. On the other hand, the CRP threshold, which was found as a predictor of in-hospital mortality by Du et al., was lower, and it was 10 mg/L [27]. Wang confirmed a positive correlation between CRP values and CT findings in the lungs in the initial stages of the disease. Their findings could give grounds for the connection between high CRP values and a more severe form of the disease [28]. Furthermore, CRP had a significantly better effect in predicting death than age, neutrophil count, and platelet count [29].

Therefore, it is essential to recognize the hyperinflammatory syndrome in COVID-19 patients, primarily over the previous quoted inflammatory parameters, and apply the anti-inflammatory therapy right on time. Corticosteroids, as anti-inflammatory drugs, have shown significant positive effects in patients with a hyperinflammatory response to SARS-CoV2 by reducing mortality, decreasing hospital stay, and increasing ventilator-free days [30, 31]. The anti-inflammatory effects of corticosteroids are proven by inducing the synthesis of anti-inflammatory proteins and, on the other hand, by inhibiting the synthesis of proinflammatory proteins [32]. It is crucial to start corticosteroid treatment at the right time and in the right patient since early administration and administration to patients with asymptomatic and milder forms of the disease may have adverse effects [33].

Elevated PCT has been detected in individuals treated for COVID-19 disease. PCT equal to or higher than 0.56 ng/mL is associated with higher mortality. Elevated PCT levels in individuals are primarily caused by bacterial coinfections, showing a good role in detecting bacterial coinfections and consequently initiating an antibiotic therapy [34]. A meta-analysis that analyzed four studies proved that an increase in PCT is associated with a five times higher risk of a more severe COVID-19 presentation (OR, 4.76; 95% CI, 2.74–8.29) [35]. Furthermore, another meta-analysis, which included over 10 thousand patients, indicated the importance of elevated PCT values as a predictor of fatal disease outcomes. The same study showed that lymphopenia, thrombocytopenia, elevated D-dimer, elevated CRP, then elevated CK, AST, ALT, LDH, and creatinine are independent predictors of deadly disease outcomes [36].

Our study proved that the concentration of D-dimer above 760 ng/mL FEU, measured on admission to the ICU, was associated with a higher risk of death during hospitalization. High values of D-dimer in COVID-19 patients are associated with local pulmonary thrombosis, which occurs as an immune-hemostatic response to prevent and limit further spread of the virus. The elevated D-dimer values exist due to a breakdown of these microthrombi [37, 38]. Elevation of D-dimer during the disease carries a higher risk of progression to severe form and mortality [3]. In particular, elevated values of D-dimer and fibrin degradation products and prolonged prothrombin time were measured at admission in the deceased subjects compared to the cured ones [39]. The study by Klok et al. [9] demonstrated that D-dimer values above 1 *μ*g/mL, measured on admission to COVID-19 treatment facilities, were associated with an eighteen times higher risk of death. Further, any increase in D-dimer values by 1 *μ*g/mL on admission is associated with an increase in the risk of death by 6%, as well as an increase in the probability (8%) of treatment with mechanical ventilation [40]. Creel-Bulos et al. showed a variation in D-dimer values in the first twenty-five days of hospitalization [41]. There is an almost linear trend of D-dimer increase in the first ten days of treatment, after which D-dimer levels are flattened. Moreover, a steeper D-dimer growth curve was observed in individuals with detected deep vein thrombosis during treatment. In contrast, differences in D-dimer growth curves were not seen in the deceased and those discharged from treatment. Zhang et al. [42] concluded that a D-dimer higher than 2 *μ*g/mL on admission could be considered a predictor of mortality during hospitalization. On the other hand, the study by Soni et al. did not prove that the values of the same parameter above 2 *μ*g/mL measured at admission were mortality predictors [43]. Still, it demonstrated that D-dimer higher than 2 *μ*g/mL during hospitalization is a mortality predictor if the highest measured values are viewed.

The literature has scarce data regarding IL-6, CRP, PCT, and D-dimer values variance in COVID-19 pneumonia and non-COVID pneumonia. Currently, the best-compared variance of mentioned parameters is between patients with COVID-19 and patients with influenza. Therefore, significantly higher CRP values on hospital admission were detected in influenza-positive subjects after comparing those groups of patients [44]. In their study, Kuang et al. have evinced the higher incidence of influenza patients detected on admission with CRP value above 10 mg/dl and PCT value above 0.5 ng/mL, compared to COVID-19 patients [45]. On the other hand, a significant increase in IL-6 level was observed in COVID-19 patients compared to patients with influenza [46]. Values of D-dimer were high on admission in both groups, COVID-19 and influenza [47]. During the 14-day monitoring of D-dimer values, a significant increase was detected in COVID-19 patients in comparison to influenza patients [48]. Considering everything, COVID-19 and influenza present a potentially life-threatening disease, and therefore, both should be treated with caution. D-dimer dynamics measurement may be used to distinguish COVID-19 infection and influenza infection.

The impact of gender on survival is still debated. Males have a higher chance for severe pneumonia, and therefore, they have a greater chance to be admitted to the ICU [49]. Potential gender-specific mechanisms modulating the course of the disease Gebhard et al. explain with a hormone-regulated expression of genes encoding for the SARS-CoV2 entry receptors ACE 2 receptor and TMPRSS2 as well as sex hormone-driven innate and adaptive immune responses and immunoaging [50]. They stressed out also elucidating the impact of gender-specific lifestyle, health behaviour, psychological stress, and socioeconomic conditions on COVID-19 [50]. Our study included 318 patients, 68.9% male and 31.1% female, admitted to ICU. These findings are in obedience to the previous study. On the contrary, we did not find a statistically significant difference between genders regarding in-hospital mortality; this statement is supported by the result in Zhou et al.'s study [11]. This can imply that biochemical parameters on admission to the ICU as predictors of in-hospital mortality should be used in all patients with the same prognostic value.

This study has several limitations that should be addressed. First, it is a single-center, retrospective study. The sample size is relatively small. Therefore, the study has limited power to detect the difference between groups. Selection bias is also presented due to the exclusion of patients without D-dimer level on admission to the ICU. Another limitation is the lack of inclusion of some data in the study, such as partial pressure of O_2_, CO_2_, BMI, etc. The reason for this is their absence in the health information system. Furthermore, we did not perform dynamic D-dimer, CRP, IL-6, and PCT measurements because of the study's retrospective design. Incorporating these data might disclose more information and give more power to our study. Unmeasured confounders such as therapy delay, previous corticosteroids use, and BMI could give us residual confounding. Finally, performing a multiple-parameter prediction model including D-dimer, CRP, IL-6, and PCT could better predict in-hospital mortality.

## 5. Conclusion

This study supported a growing body of literature regarding hyperinflammatory syndrome and diffuse microvascular thrombosis as predictors of poor clinical outcomes in COVID-19 patients. Proper and on-time differentiation patients with lower survival chances may be crucial for starting anti-inflammatory therapy such as corticosteroids, which may reduce in-hospital mortality. In particular, IL-6 ≥ 74.98 pg/mL, CRP ≥ 81 mg/L, PCT ≥ 0.56 ng/mL, and D-dimer ≥ 760 ng/mL on admission to the ICU could effectively predict in-hospital mortality in COVID-19 patients. Using these laboratory parameters single or in combination may help identify patients with lower survival chances and, on time, improve further treatment. Further prospective multicenter studies are necessary to confirm our findings.

## Figures and Tables

**Table 1:** Age, comorbidities, laboratory parameters, and CT score of patients participated in the study. Results are expressed in *n* (%) and median (IQR).

	Total (*n* = 318)	No survivor (*n* = 195)	Survivor (*n* = 123)	*p* value
Age (years)	69 (60-77)	72 (64-79)	63 (51-73)	**<0.001**
Males, *n* (%)	219 (68.9)	130 (59.4)	89 (40.6)	0.286
Females, *n* (%)	99 (31.1)	65 (65.7)	34 (34.3)	

*Comorbidities*				
Hypertension, *n* (%)	223 (70.1)	140 (71.8)	83 (67.5)	
Diabetes mellitus, *n* (%)	100 (31.4)	54 (27.7)	46 (37.4)	
Coronary disease, *n* (%)	62 (19.5)	37 (19)	25 (20.3)	
Obesity, *n* (%)	40 (12.6)	21 (10.8)	19 (15.4)	
Cardiomyopathy, *n* (%)	27 (8.5)	18 (9.2)	9 (7.3)	
COPD, *n* (%)	19 (6)	13 (6.7)	6 (4.9)	
Asthma, *n* (%)	14 (4.4)	9 (4.6)	5 (4.1)	

*Laboratory parameters*				
IL-6 (pg/L)	110.8 (44.1-399.6)	160.7 (71.4-812.3)	66.8 (29.7-239)	**<0.001**
CRP (mg/L)	88 (53.8-191.5)	103.4 (61.1-210.1)	75.5 (41.7-177.2)	**<0.001**
Lymphocyte (%)	0.7 (0.5-1.1)	0.7 (0.5-1)	0.8 (0.5-1.2)	0.063
Serum ferritin (*μ*g/L)	822 (415.5-1478)	766.5 (374-1409.2)	760.5 (306.7-1416)	0.673
PCT (ng/mL)	1.1 (0.2-9)	3.27 (0.8-17.9)	0.2 (0.1-0.7)	**<0.001**
D-dimer (ng/mL)	829 (497-2759.5)	1121.5 (594-3212.2)	666 (353.5-1317)	**<0.001**
Platelet count (×10^9^/L)	225 (161.5-303)	204.5 (146-281)	234 (179.7-339.5)	0.022
INR	1.1 (1-1.3)	1.1 (1-1.3)	1.1 (1-1.2)	0.341
aPTT (s)	25.6 (22.5-30.3)	26 (22.7-30.3)	24.6 (22.4-28.4)	0.073
Fibrinogen (g/L)	4.1 (3.5-4.9)	4.1 (3.5-5)	4.2 (3.4-5.1)	0.921
Albumin (g/L)	32 (29-35)	31 (27.5-33)	35 (32-38)	**<0.001**
*CT score*	17 (5-22)	17 (1.5-22)	17 (8-22)	0.96

**Table 2:** C-statistic of IL-6, CRP, PCT, and D-dimer to predict mortality in patients with COVID-19.

Predictor value	*C*-index	95% confidence interval
IL-6	0.64	0.57–0.71
CRP	0.62	0.56–0.69
PCT	0.77	0.71–0.83
D-dimer	0.64	0.57–0.7

## Data Availability

The data used to support the findings of this study are available from the corresponding author (AH) upon request.

## Abbreviations

SARS-CoV-2: Severe acute respiratory syndrome coronavirus 2
COVID-19: Coronavirus disease 2019
ARDS: Acute respiratory distress syndrome
DIC: Disseminated intravascular coagulation
MODS: Multiorgan dysfunction syndrome
LMWH: Low molecular weight heparin
ICU: Intensive Care Unit
IL-6: Interleukin 6
CRP: C-reactive protein
PCT: Procalcitonin
CT: Computed tomography
RT-PCR: Reverse transcription-polymerase chain reaction
BMI: Body mass index
COPD: Chronic obstructive pulmonary disease
PT: Prothrombin time
aPTT: Activated partial thromboplastin time
IQR: Interquartile range
Sn: Sensitivity
Sp: Specificity
IL-1, IL-2, IL-7, IL-8, and IL-12: Interleukins 1, 2, 7, 8, and 12
IFN: Interferon
MCP-1: Membrane cofactor protein 1
TNF-*α*: Tumor necrosis factor *α*
CK: Creatine kinase
AST: Aspartate Aminotransferase
ALT: Alanine Aminotransferase
LDH: Lactate dehydrogenase
ACE 2: Angiotensin converting enzyme 2
TMPRSS2: Transmembrane Serine Protease 2
